# The lack of increases in circulating endothelial progenitor cell as a negative predictor for pathological response to neoadjuvant chemotherapy in breast cancer patients

**DOI:** 10.1038/s41698-017-0006-1

**Published:** 2017-04-17

**Authors:** Sunao Tanaka, Takayuki Ueno, Hiroshi Ishiguro, Satoshi Morita, Masakazu Toi

**Affiliations:** 1 Department of Breast Surgery, Graduate School of Medicine and Faculty of Medicine Kyoto University, 54 Shogoin-Kawahara-cho, Sakyo-ku, Kyoto 606-8507 Japan; 2 Department of Breast Surgery, School of Medicine Kyorin University, 6-20-2 Shinkawa, Mitaka, Tokyo 181-8611 Japan; 3 Department of Target Therapy Oncology, Graduate School of Medicine and Faculty of Medicine Kyoto University, 54 Shogoin-Kawahara-cho, Sakyo-ku, Kyoto 606-8507 Japan; 4 Department of Biomedical Statistics and Bioinformatics, Graduate School of Medicine and Faculty of Medicine Kyoto University, 54 Shogoin-Kawahara-cho, Sakyo-ku, Kyoto 606-8507 Japan

## Abstract

Circulating endothelial progenitor cells are a potential surrogate marker for angiogenesis. Little is known about the alteration of circulating endothelial progenitor cell counts during neoadjuvant chemotherapy. Our goal was to reveal the alteration in CEP counts in association with response to neoadjuvant chemotherapy in patients with breast cancer. We measured the number of circulating endothelial progenitor cells (CD31^+^CD34^+^CD133^+^CD45^dim^) by four-color flow cytometry using blood samples from 57 patients with breast cancer who received neoadjuvant chemotherapy (5-fluorouracil + epirubicin + cyclophosphamide (FEC), docetaxel + cyclophosphamide (TC), cisplatin + docetaxel (TP)). There was no significant difference in the baseline circulating endothelial progenitor cell counts with respect to the clinical and pathological background factors. Circulating endothelial progenitor cell counts increased after the initiation of chemotherapy (pre-1st vs. pre-2nd cycle, *p* = 0.0035; pre-1st vs. pre-4th cycle, *p* = 0.047). An increase of circulating endothelial progenitor cell counts from pre-1st to pre-2nd cycle was associated with pCR (*p* = 0.013 for *χ* ^2^ test). A multivariate analysis, including subtype, and clinical response showed that the lack of circulating endothelial progenitor cell increases from pre-1st to pre-2nd cycle was an independent negative predictor of pCR (*p* = 0.002). Our data suggest that alterations in circulating endothelial progenitor cell counts are associated with treatment response. The circulating endothelial progenitor cell count could be a useful biomarker for monitoring chemotherapeutic response.

## Breast cancer: Circulating cell counts predict chemo response

Breast cancer patients do better after preoperative chemotherapy if their numbers of circulating endothelial progenitor cells (CEPs) go up. A team from Japan led by Takayuki Ueno from the Kyorin University School of Medicine in Tokyo studied 57 women who underwent neoadjuvant chemotherapy to shrink the size of their tumors before surgery. The researchers measured the number of CEPs—cells that derive from the bone marrow and can help build new blood vessels around tumors—both before and during chemotherapy. They found no link between overall CEP counts and treatment response. However, patients whose CEP numbers went up between cycles of chemotherapy had better outcomes. The authors suggest that CEP counts could be used as a diagnostic tool for predicting responses to neoadjuvant chemotherapy, although larger studies are needed to confirm their initial findings

## Introduction

Angiogenesis is a complex process that relies on the harmonization of activities in different cell types. Endothelial progenitor cells, mature endothelial cells, pericytes, fibroblasts, and immune mediators express a number of cytokines and growth factors that interact with each other or with extracellular matrix components to affect endothelial cell migration, proliferation, tube formation, and vasculature stabilization. Circulating endothelial progenitor cells (CEPs) are mobilized from the bone marrow and subsequently home to sites of tumor vascularization. They differentiate into endothelial cells, resulting in angiogenesis.^1^ The number of CEPs is reportedly altered by different factors, such as growth factors; hormones; menstrual cycle; and life habits, such as smoking and exercise.^2–7^

It has been suggested that circulating endothelial cells (CECs) and CEPs could serve as surrogate markers to define the optimal biological dose of anti-angiogenesis therapies.^8^ We have reported that CEC counts determined using CellSearch system altered during chemotherapy in patients with breast cancer and that these counts were associated with the therapeutic response.^9, 10^ During chemotherapy, a remarkable mobilization of CEPs from the bone marrow into the circulation occurs presumably as an adaptive response to chemotherapy-induced endothelial damage in order to replace the damaged cells.^11^ However, alterations in CEP counts during neoadjuvant chemotherapy (NAC) and their association with the therapeutic response largely remain to be elucidated.

Early identification of patients who benefit from NAC is critical for the optimization of chemotherapy and for avoiding any serious side effects in non-responders. The aim of this study was to investigate changes in CEP counts in patients with primary breast cancer, receiving NAC, in order to examine the association between CEP kinetics and therapeutic response.

## Results

### Baseline characteristics of patients and CEP count

Baseline blood samples from 57 patients with primary breast cancer were collected prior to NAC (Supplementary Fig. 1). The CEP counts ranged from 0 to 147 cells per 1 × 10^5^ mononuclear cells and the distribution was shown in Supplementary Fig. 2. Table 1 shows clinical and pathological characteristics of patients and their CEP counts. No significant difference in baseline CEP counts was observed with respect to age; menopausal status; tumor size; nodal status; and pathological factors, including hormone receptors and human epidermal growth factor receptor type 2 (HER2) expression.

**Table 1:** Patient clinical and pathological characteristics and CEP counts at baseline (*n* = 57)

Characteristic		No. of patients	CEP median	25th–75th percentile	*p*-value
*Age*	30s	13	34	25.5–49.5	0.87
	40s	15	39	23–54	
	50s	18	34	21.75–47.25	
	60s	11	41	24–58	
*T*	1	12	34.5	27.5–50.5	0.75
	2	30	36	21.75–46.75	
	3	12	42.5	25–65.5	
	4	3	23	21–100	
*N*	0	27	38	24–49	0.57
	1	28	36	23.25–57	
	2	1	49	–	
	3	1	21	–	
*Hormone receptor*					
ER	positive	31	38	24–54	0.41
	negative	26	32	22.5–49.5	
PR	positive	25	32	22.5–49.5	0.26
	negative	32	39	27.5–56.25	
HER2	positive	18	34	24–49.5	0.97
	negative	39	36	23–58	
*Menopausal status*					
premenopausal		29	31.5	22–41	0.85
postmenopausal		26	40	22.5–52.75	
hysterectomy		2	31.5	22–41	
*chemotherapy regimen*					
anthracycline-based (followed by taxane <Trastuzumab>)		24(13<1>)	34	24–48.25	0.75
taxane-based (followed by anthracycline)		22(7)	39	23–60.5	
platinum-based (followed by anthracycline)		11(4)	34	23–66	
*pathological response*					
pCR		19	31	20–49	0.40
non-pCR		38	36	23.75–60	
*clinical response*					
responder		46	36	22.75–51.75	0.50
non-responder		11	36	29–53	

*ER* estrogen receptor, *PR* progesterone receptor, *HER2/neu* human epidermal growth factor receptor type 2, *pCR* pathological complete response

### CEP counts and pathological response to chemotherapy

**Fig. 1:**
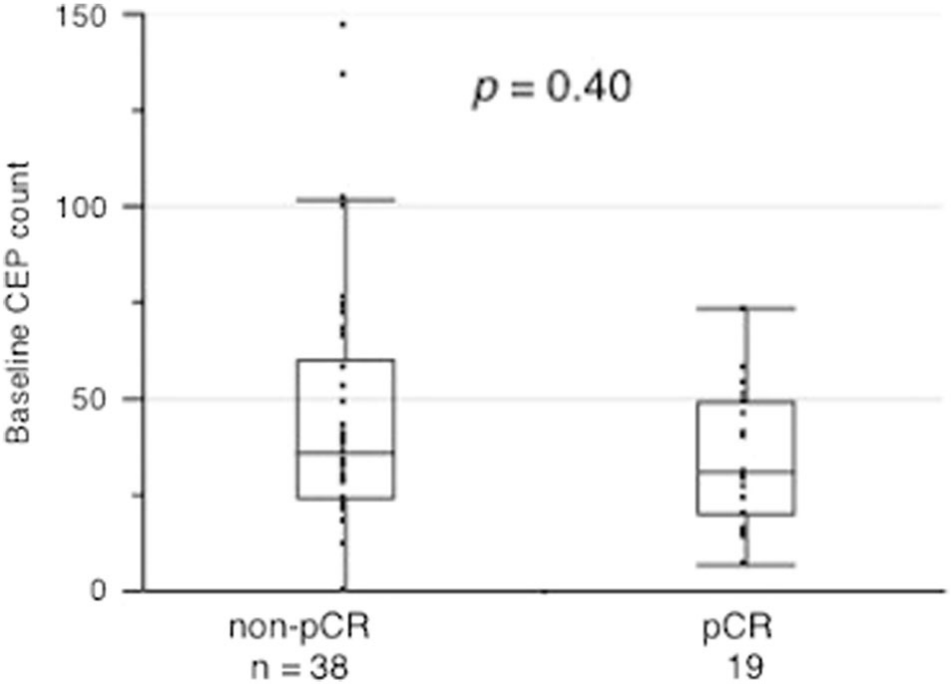
Comparison of baseline CEP counts between non-pCR and pCR groups. The y-axis indicates the baseline CEP count. There was no significant difference in CEP counts between the non-pCR and pCR groups (*p* = 0.40, Mann—Whitney test : two-sided)

Baseline CEP counts were examined according to the pathological response to NAC. There was no significant difference in baseline CEP counts between the pCR and non-pCR groups (*p* = 0.40; Fig. 1).

### CEP alterations during chemotherapy

**Fig. 2:**
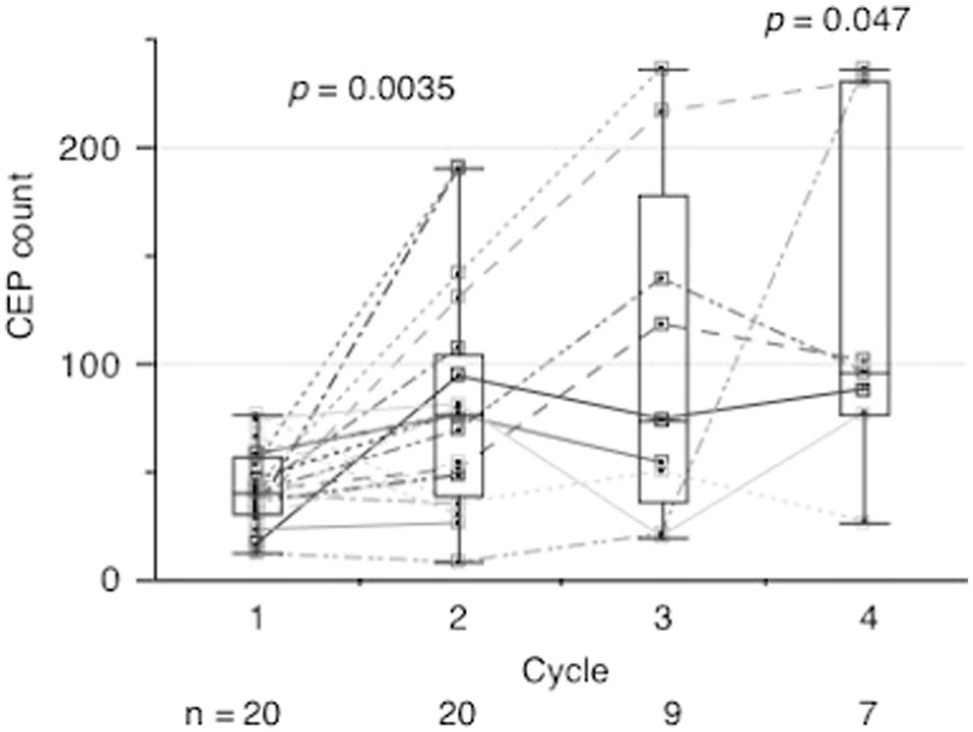
Alteration in CEP counts during neoadjuvant chemotherapy. The y-axis indicates CEP counts. The number of CEPs increased during neoadjuvant chemotherapy (pre-1st vs. pre-2nd cycle, *p* = 0.0035; pre-1st vs. pre-4th cycle, *p* = 0.047, Wilcoxon signed-rank test : two-sided)

**Fig. 3:**
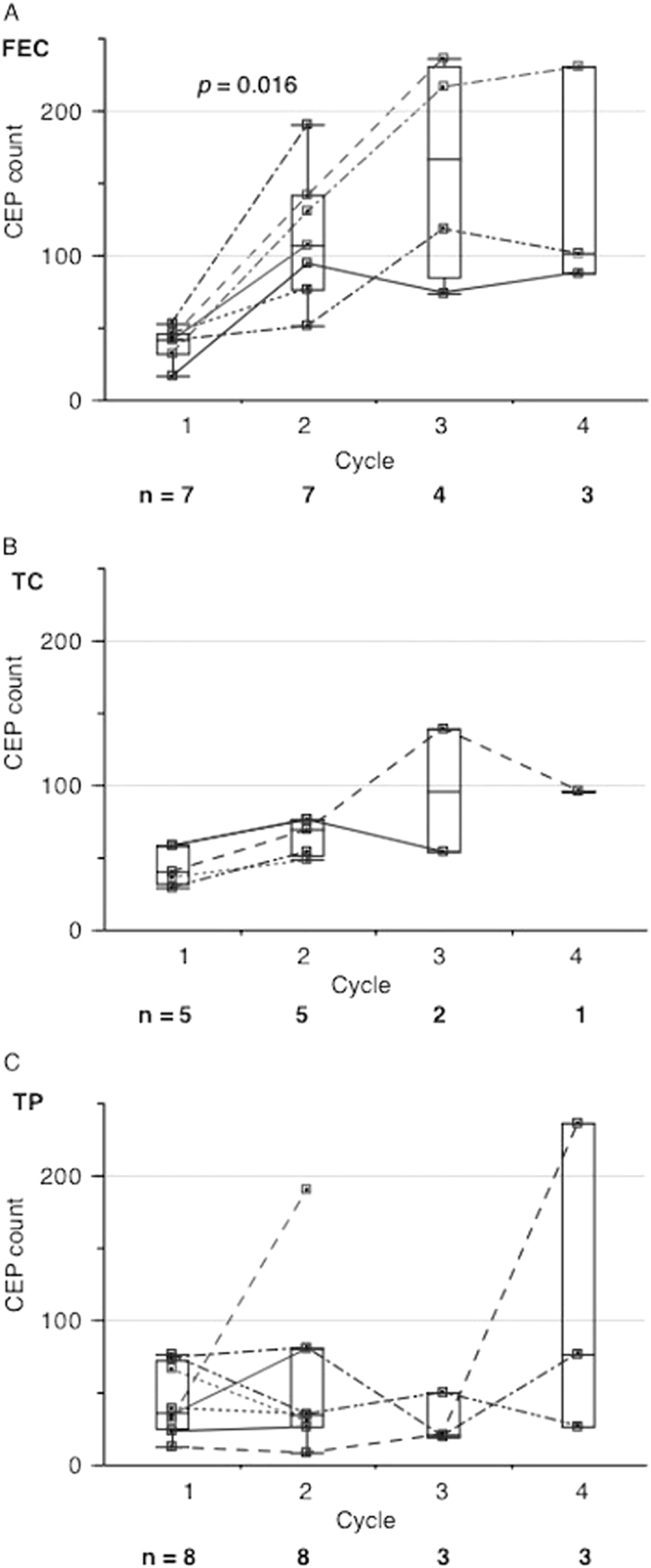
Alteration in CEP counts during neoadjuvant chemotherapy with respect to regimen (A: FEC, B: TC, C: TP). The y-axis indicates CEP counts. In patients receiving the anthracycline-based regimen, the number of CEPs increased from pre-1st to pre-2nd cycle of chemotherapy (*p* = 0.016, Wilcoxon signed-rank test : two-sided)

Alterations in CEP counts were monitored in 20 of the 57 patients (Supplementary Fig. 1 and Supplementary Table 1). No significant difference in baseline CEP counts was observed with respect to clinical and pathological characteristics in the 20 patients (Supplementary Fig. 3). The number of CEPs increased during chemotherapy (pre-1st vs. pre-2nd cycle, *p* = 0.0035; pre-1st vs. pre-4th cycle, *p* = 0.047; Fig. 2). CEP alterations were analyzed according to the chemotherapy regimens. Patients who received the anthracycline-based regimen showed increased CEP counts from pre-1st to pre-2nd cycle of chemotherapy (*p* = 0.016; Fig. 3a), whereas those who received taxane-based or platinum-based regimens did not show any increase in the counts (Figs. 3b and 3c).

### Alterations in CEP counts and pathological response to chemotherapy

**Fig. 4:**
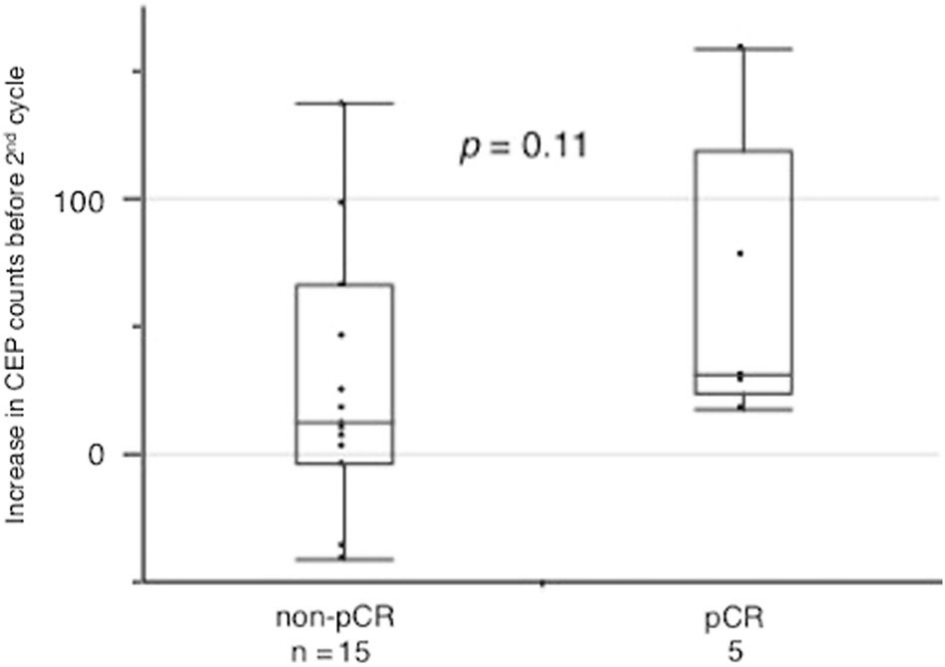
The increase in CEP counts between 1st and 2nd cycle of chemotherapy according to the pCR status. The y-axis indicates the increase in CEP counts between 1st and 2nd cycle. Patients with pCR tended to showed a greater increase in CEP counts (*p* = 0.11, Mann—Whitney test : two-sided)

**Fig. 5:**
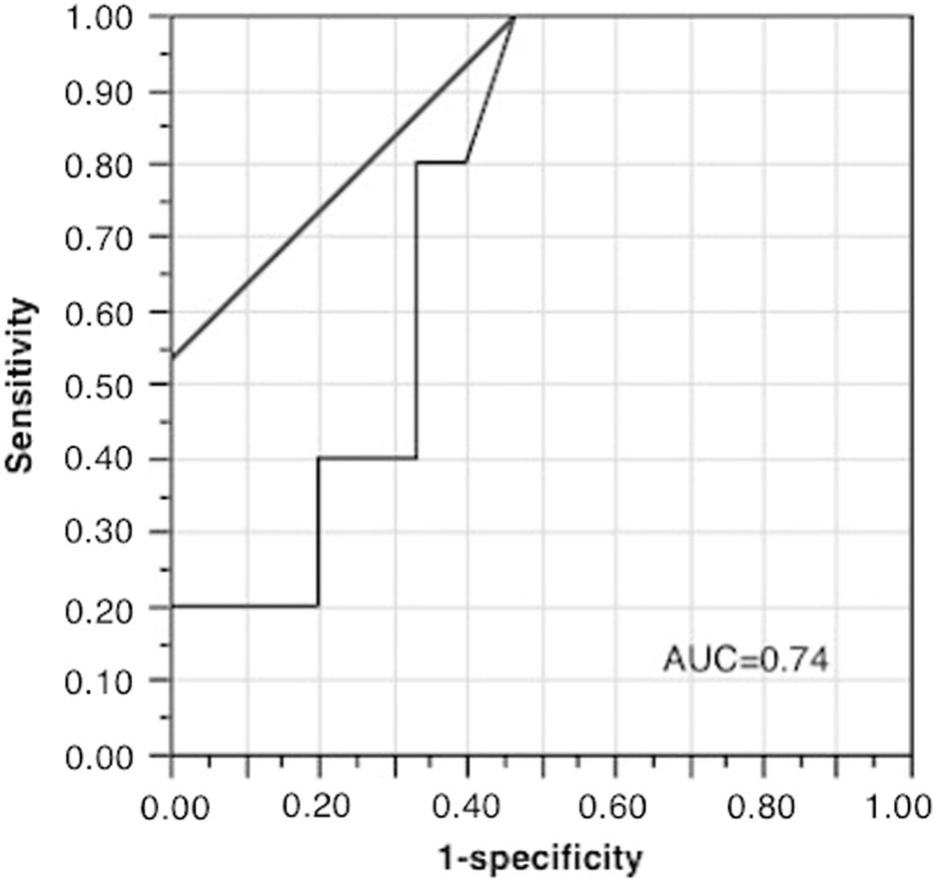
Receiver operating characteristic (ROC) curve for pCR to determine the CEP increase cut-off. The x-axis indicates 1-specificity, and the y-axis indicates sensitivity. The area under the ROC curve was 0.74. It gave the CEP increase cut-off as 15 cells (AUC = 0.74)

Although CEP counts increased from pre-1st to pre-2nd cycle of chemotherapy in the entire patient population (Fig. 2), patients with pCR tended to have a greater increase (*p* = 0.11; Fig. 4). Thus, we hypothesized that a greater CEP increase was associated with pathological response and created a receiver operating characteristic (ROC) curve based on the increase in CEP counts to distinguish between pCR and non-pCR. The area under the ROC curve was 0.74 and gave a CEP increase cut-off value of 15 using the Youden Index (Fig. 5).^12^ According to this cut-off value, all the patients with a pCR showed an increase in CEPs, while less than half of the non-pCR patients did (*p* = 0.013; Table 2). This cut-off value gave the sensitivity of 100% and the specificity of 55% for pCR (Fig. 5).

**Table 2:** CEP increases and pathological response

	non pCR	pCR
CEP increase	7	5
No increase	8	0

*CEP* circulating endothelial progenitor cells, *pCR* pathological complete response.

*p* = 0.013 ^*^ *p* < 0.05 was considered as statistically significant

To investigate the predictive value of the CEP increase for pCR, the logistic regression analysis was applied and included clinical and pathological factors (Table 3). In the univariate analysis, the CEP increase and clinical response were significantly associated with pCR (*p* = 0.01 and 0.04, respectively), while the subtype was marginally associated (*p* = 0.06; Table 3 and Supplementary Table 2). Multivariate analysis was performed using factors with *p* < 0.1 in the univariate analysis (model 1). The lack of CEP increases was an independent negative predictor for pCR (*p* = 0.002) in addition to clinical response (*p* = 0.01; Table 3). Because the chemotherapy regimen was associated with the CEP increase (Fig. 3a, b, and c), another model including the chemotherapy regimen was tested in order to examine whether the CEP increase was an independent predictor. Indeed, the lack of CEP increases remained an independent negative predictor after adjusting for the regimen type (*p* = 0.019).

**Table 3:** Univariate and multivariate analysis of pCR (logistic regression analysis)

Parameters	Univariate (*p*-value)	Multivariate (*p*-value)	
		Model 1	Model 2
Age	0.58	–	–
Menopausal status	0.35	–	–
*T*	0.42	–	–
*N*	0.18	–	–
Grade	0.6	–	–
subtype	0.06	0.079	0.11
regimen	0.51	–	1
Clinical response	0.04	0.01*	0.096
CEP counts (baseline)	0.52	–	–
CEP increase	0.01	0.002*	0.019*

*CEP* circulating endothelial progenitor cells, *pCR* pathological complete response.

*p < 0.05 was considered as statistically significant

## Discussion

In the current study, we investigated changes in CEP counts during NAC and found that CEP counts increased from pre-1st to pre-2nd cycle of chemotherapy. In particular, the lack of increases in CEPs from pre-1st to pre-2nd cycle was an independent negative predictor for pCR, suggesting that monitoring CEP counts could be useful when deciding on treatment continuation. Although the specificity of CEP increases for pCR was 55%, the negative predictive value was 100%, thus we considered that the lack of CEP increases could have a negative predictive value for pCR. Because CEP mobilization into the circulation has been suggested to be an adaptive response to the chemotherapy-induced endothelial damage, it is reasonable to assume that an early tumor response leads to endothelial damage, resulting in an early increase in CEPs.^11^ Indeed, it has been reported that tumors that achieve pCR with NAC tend to show an early response, which is in good agreement with our theory.^13^ Notably, none of the patients without CEP increase exhibited pCR in our study. A larger study is clearly needed to confirm our results.

The relationship between CEP counts and response to chemotherapy has been reported in several studies. A study by Sakamori *et al.* showed that in non-small-cell lung cancer, the increase in CEP counts before the 2nd cycle of chemotherapy was higher in responders than in nonresponders,^14^ which agrees with our results. Similar to our definition, they defined CEP as CD31^+^CD34^+^CD133^+^CD45^−^, and their patients received mostly a platinum-based regimen, similar to one of the regimens used in this study. In contrast, another study showed that CEPs decreased in responders and increased in nonresponders with multiple myeloma.^15^ However, they defined CEP as CD34^+^VEGFR2^+^CD45^−/dim^, different from our definition, and their patients received bortezomib and dexamethasone. Hence, this discrepancy may stem from differences in CEP definitions, therapeutic agents, and malignancy types.

Clinically, the standardization of the CEP definition is critical. Unique markers have not yet been reported to specifically identify CEP, and endothelial projenitor cells express a variety of antigens, such as CD13, CD31, CD34, CD105, CD117, CD133, CD144, CD146, and VEGFR2.^16^ Some researchers used CD34^+^VEGFR2^+^ for CEP enumeration, but CD34 and VEGFR2 are also expressed by mature CECs.^17^ It has been reported that CD45^dim^ and CD133^+^ populations include CEPs and not CECs.^16^ In the present study, we used multiparametric flow cytometry in combination with CD31, CD34, CD45, and CD133 to detect CEP populations based on these findings. Nonetheless, the validation and standardization of procedures to detect CEPs is necessary in order to utilize CEP as a reliable biomarker in clinical applications.

Interestingly, the changes in CEP counts during NAC differed according to regimens. These counts significantly increased in patients who received anthracycline-based regimen but not in those who received the other regimens. It has been demonstrated that certain chemotherapy drugs, such as taxane and fluorouracil, rapidly induce CEP mobilization within hours of administration as well as subsequent tumor homing, while others, such as gemcitabine, cisplatin, and doxorubicin, do not.^18, 19^ Because we did not examine the alteration in CEP counts within hours of drug administration, it is conceivable that a rapid increase in CEPs with the taxane-based regimen was missed. Doxorubicin has been reported to induce apoptosis in endothelial cells with generation of reactive oxygen species, disrupting the balance between nitric oxide and superoxide.^20^ Previous studies have shown that increased redox cycling of the doxorubicin semiquinone radical leads to increased oxidative stress and endothelial cell apoptosis via caspase-dependent mechanisms.^21^ Several clinical studies have also shown that anthracyclines can induce apoptosis in endothelial cells, and the repair processes might result in overexpression of vascular endothelial growth factor (VEGF).^22, 23^ The results of these studies might account for the increase in CEPs during the anthracycline-based regimen observed in our study. Trastuzumab with docetaxel was given following FEC in a patient with HER2-positive disease, and so CEP was not monitored during trastuzumab because CEP was monitored only during FEC (the 1st regimen). It is important to examine the changes in CEP during trastuzumab in the future study.

Another factor to be considered during NAC is the use of granulocyte-colony stimulating factor (G-CSF). G-CSF is a well-known mobilizing factor of CEP. It has been reported that G-CSF induces a rapid spike in CEP counts after 4 h in mice and that CEPs induced by G-CSF in humans have reduced migratory responses to VEGF and stromal cell-derived factor-1.^17, 24^ However, a comparison between patients who received G-CSF during chemotherapy and those who did not showed no differences in CEP increase in our study (Supplementary Fig. 4). This result could be due to the short half-life (2–4 h) of subcutaneously administered G-CSF^25^ and the difference in time points between G-CSF administration and CEP measurement. Notably, pegylated G-CSF was not used in our study because it was not approved for use during the study period. A further study is needed to examine the alterations in CEP counts using pegylated G-CSF during chemotherapy.

There are several limitations to our study. The main limitation was the small sample size. In order to be clinically useful and relevant, the chemotherapeutic regimens were restricted to standard FEC, TC, and TP regimens. In addition, blood collection for this study was not performed before the 2nd cycle in some patients because of the hospital blood collection system, which resulted in a smaller sample size. At the time of the study, blood samples for research could be collected only from inpatients, which made this limitation. Thus, the results in this study could be biased and validation studies with a larger sample size are needed to confirm our results. Another limitation is the use of only three types of chemotherapy regimens in this study. As suggested by the current and previous studies, changes in CEP counts might differ according to the chemotherapy regimen. Although our study showed that the CEP increase was associated with pCR independent of the regimen, the possibility that different regimens might affect CEP counts differently remains. The lack of a standardized CEP definition is another limitation. Different definitions may give different clinical utilities and, thus, the standardization of CEP measurement is urgent for development of optimal monitoring markers, which require further studies.

In conclusion, the increase in CEP counts was observed during NAC in patients with breast cancer, and the lack of CEP increases from pre-1st to pre-2nd cycle was an independent negative predictor for pCR. Therefore, we concluded that CEP counts may be useful for monitoring response to NAC. A further study with a larger sample size is required to confirm our results.

## Materials and methods

### Study population

Blood samples at baseline (pre-cycle 1) were collected from 57 patients with primary breast cancer who received NAC at the Kyoto University Hospital from December 2007 to November 2011. Blood samples were also collected during NAC. (pre-cycle 2 : *n* = 20, pre-cycle 3 : *n* = 9, pre-cycle 4 : *n* = 7) Blood (10 mL) was drawn into a CellSave preservative tube (Veridex, North Raritan, NJ, USA). NAC included anthracycline-based, taxane-based, and platinum-based regimens. The anthracycline-based regimen comprised four cycles of FEC (500 mg/m^2^ 5-fluorouracil, 100 mg/m^2^ epirubicin, and 500 mg/m^2^ cyclophosphamide) tri-weekly (*n* = 24). The taxane-based regimen comprised four cycles of TC (75 mg/m^2^ docetaxel and 600 mg/m^2^ cyclophosphamide) tri-weekly (*n* = 22). The platinum-based regimen comprised four cycles of TP (75 mg/m^2^ cisplatin and 75 mg/m^2^ docetaxel) tri-weekly (*n* = 11) (Supplementary Fig. 1). Trastuzumab in combination with docetaxel (75 mg/m^2^) was given in one patient with HER2-positive disease, following FEC. Clinical response to chemotherapy was assessed according to the Response Evaluation Criteria in Solid Tumors version 1.0.

### Flow cytometry

Mononuclear cells were isolated by density centrifugation using Ficoll-Paque Plus gradients (GE Healthcare Bio-Science, Sweden) according to the manufacturer’s protocol. Mononuclear cells were rinsed twice with phosphate-buffered saline (PBS) and incubated with allophycocyanin-labeled monoclonal mouse antihuman CD31 (clone AC128), fluorescein isothiocyanate-conjugated monoclonal mouse antihuman CD34 (clone AC136), phycoerythrin-conjugated monoclonal mouse antihuman CD133 (clone AC133) (all antibodies from Miltenyi Biotec, Bergisch Gladbach, Germany), and peridinin chlorophyll protein-conjugated monoclonal mouse antihuman CD45 (clone 2D1; BD Biosciences, Sweden) antibodies. Samples were resuspended in 400 μL of PBS, and CEPs were measured using a FACSCalibur flow cytometer (Becton, Dickinson and Company, NJ, USA). Fluorochrome- and isotype-matched controls were used to normalize appropriate regions. CEPs were defined as CD31^+^CD34^+^CD133^+^CD45^dim^ cells.^16^ Data were expressed as the number of CEP cells per 1 × 10^5^ mononuclear cells.^5, 16, 26^

### Pathological analysis

Tumor core biopsy specimens before NAC were used for pathological examination. Tumor grade was assessed according to the Scarff—Bloom—Richardson grading system. Estrogen receptor (ER) and progesterone receptor (PR) statuses were defined as positive for tumors having 1% or more positive tumor cells using SP1 and 1E2 antibodies (both from Roche Diagnostics, Tokyo, Japan), respectively. HER2 positivity was determined by strong expression (3+) of HER2 using the HercepTest (Dako, Denmark) or by a HER2:CEP17 ratio > 2.2 using fluorescence in situ hybridization (FISH). Tumor subtypes were identified based on ER, PR, and HER2 expression. Tumors that were ER+ and/or PR+ and HER2− were considered luminal, those that were ER+ and/or PR+ and HER2+ were considered luminal/HER2, those that were ER−, PR−, and HER2+ were considered HER2, and those that were ER−, PR−, and HER2− were considered triple negative.

The pathological response was assessed using surgical specimens; a pathological complete response (pCR) was defined as no residual invasive tumor cells in mammary glands and lymph nodes.

### Statistical analysis

Statistical analysis was performed using the Wilcoxon signed-rank test for comparisons of CEP counts between cycles of chemotherapy, Mann–Whitney test for comparisons between pCR and non-pCR, and Kruskal—Wallis test for comparisons among three or more groups. A *χ* ^2^ test was used for comparisons between CEP increase and pCR. Univariate and multivariate logistic regression analyses were performed to examine the association between pCR and clinical or pathological factors. All analyses were performed using JMP version 9.0.0 software (SAS Institute, Inc., NC, USA). All *p*-values were two-sided, and *p* < 0.05 was considered as significant.

### Ethical approval

The study was approved by the Institutional Ethical Committee of Kyoto University (E396).

### Informed consent

Written informed consent was obtained from all patients included in the study before enrollment.

## Electronic supplementary material

Supplementary Figure 1

Supplementary Figure 2

Supplementary Figure 3

Supplementary Figure 4

Supplementary Table 1

Supplementary Table 2

## Electronic supplementary material

Supplementary Information accompanies the paper on the *npj Precision Oncology* website (doi:10.1038/s41698-017-0006-1).
