# Supplementary figures and images for: The lack of increases in circulating endothelial progenitor cell as a negative predictor for pathological response to neoadjuvant chemotherapy in breast cancer patients
Source: NPJ Precis Oncol. 2017 Apr 17;1:6. doi: 10.1038/s41698-017-0006-1 (PMC5871813; doi:10.1038/s41698-017-0006-1)

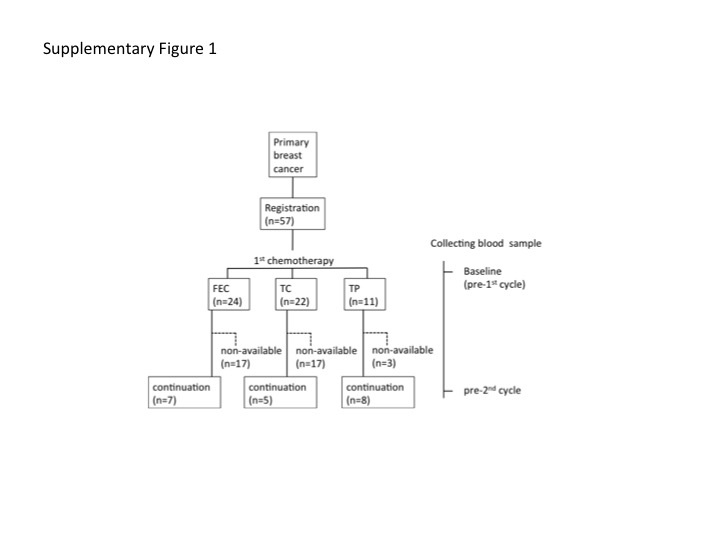

Supplement: Supplementary file 1 — Supplementary Figure 1 [file 41698_2017_6_MOESM1_ESM.jpg]

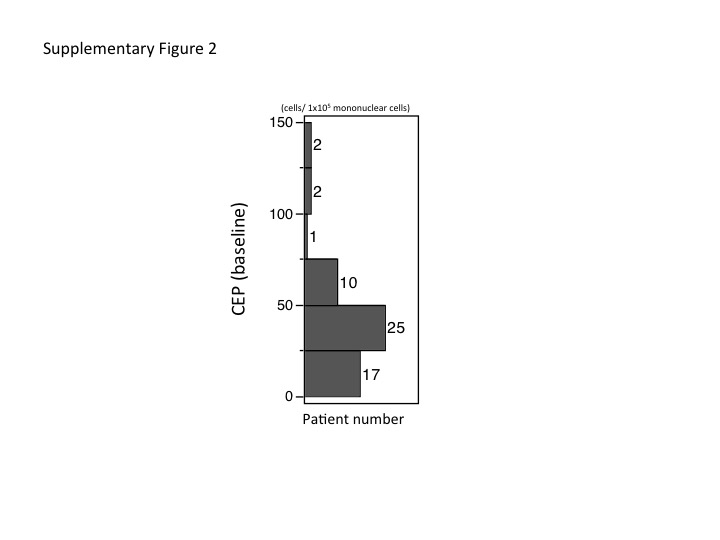

Supplement: Supplementary file 2 — Supplementary Figure 2 [file 41698_2017_6_MOESM2_ESM.jpg]

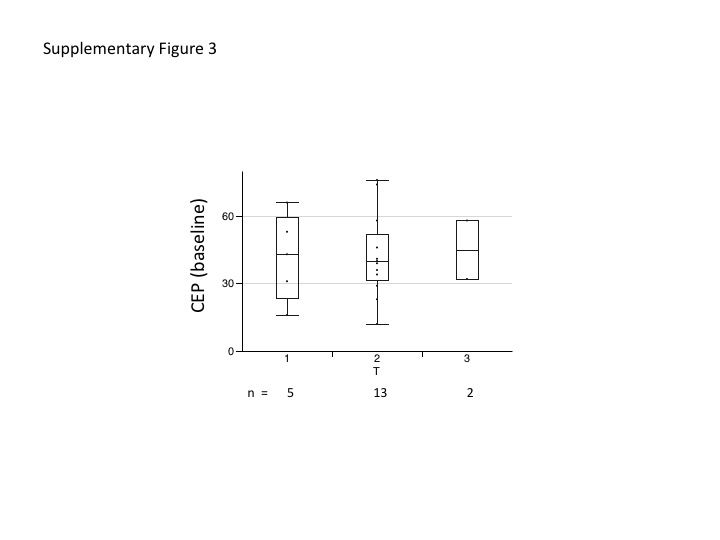

Supplement: Supplementary file 3 — Supplementary Figure 3 [file 41698_2017_6_MOESM3_ESM.jpg]

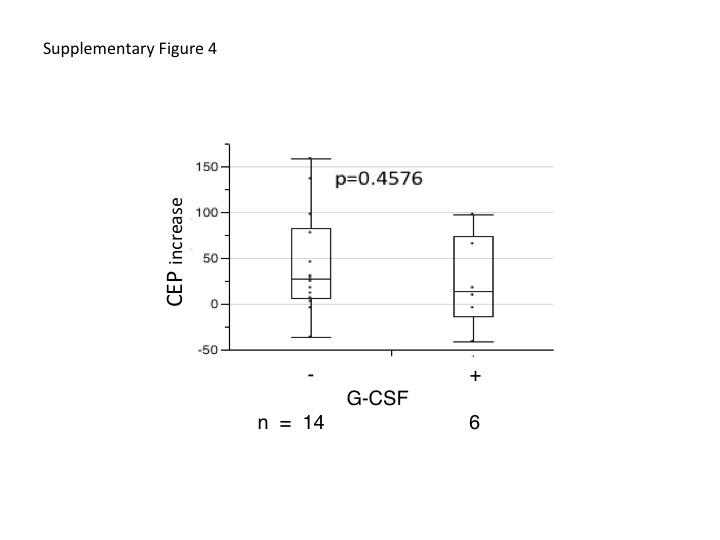

Supplement: Supplementary file 4 — Supplementary Figure 4 [file 41698_2017_6_MOESM4_ESM.jpg]
