# Supplementary material for: The lack of increases in circulating endothelial progenitor cell as a negative predictor for pathological response to neoadjuvant chemotherapy in breast cancer patients
Source: NPJ Precis Oncol. 2017 Apr 17;1:6. doi: 10.1038/s41698-017-0006-1 (PMC5871813; doi:10.1038/s41698-017-0006-1)
Supplement: Supplementary file 5 — Supplementary Table 1 [file 41698_2017_6_MOESM5_ESM.docx]

**Supplementary Table 1.** Clinical and pathological characteristics and CEP counts in patients whose CEP counts were monitored during neoadjuvant chemotherapy (n = 20)

| **Characteristic** | | **No. of patients** |
| --- | --- | --- |
| **Age** | **Median** | 53 |
|  | **Range** | 33-69 |
| **T** | **1** | 5 |
|  | **2** | 13 |
|  | **3** | 2 |
| **N** | **0** | 11 |
|  | **1** | 9 |
| **Hormone receptor** | |  |
| **ER** | **positive** | 9 |
|  | **negative** | 11 |
| **PR** | **positive** | 7 |
|  | **negative** | 13 |
| **HER2** | **positive** | 4 |
|  | **negative** | 16 |
| **menopausal status** | |  |
| **premenopausal** | | 7 |
| **postmenopausal** | | 12 |
| **hysterectomy** | | 1 |
| **chemotherapy regimen** | |  |
| **anthracycline-based (followed by taxane < + Trastuzumab>))** | | 7 (5 <1>) |
| **taxane-based (followed by anthracycline)** | | 5 (2) |
| **platinum-based (followed by anthracycline)** | | 8 (3) |
| **pathological response** | |  |
| **pCR** | | 5 |
| **non-pCR** | | 15 |
| **clinical response** | |  |
| **responder** | | 14 |
| **non-responder** | | 6 |

ER, estrogen receptor; PR, progesterone receptor; HER2, human epidermal growth factor receptor type 2; pCR, pathological complete response
