# Supplementary material for: The lack of increases in circulating endothelial progenitor cell as a negative predictor for pathological response to neoadjuvant chemotherapy in breast cancer patients
Source: NPJ Precis Oncol. 2017 Apr 17;1:6. doi: 10.1038/s41698-017-0006-1 (PMC5871813; doi:10.1038/s41698-017-0006-1)
Supplement: Supplementary file 6 — Supplementary Table 2 [file 41698_2017_6_MOESM6_ESM.docx]

**Supplementary Table 2**

| **Subtype** | **pCR rate** | **CEP increase** |
| --- | --- | --- |
| ER+HER2- | 43% (3/7) | 71% (5/7) |
| ER+HER2+ | 0% (0/2) | 100% (2/2) |
| ER-HER2+ | 50% (1/2) | 100% (2/2) |
| ER-HER2- | 11% (1/9) | 33% (3/9) |
